# Novel purification method and antibiotic activity of recombinant *Momordica charantia* MAP30

**DOI:** 10.1007/s13205-016-0590-8

**Published:** 2017-04-07

**Authors:** Ching-Dong Chang, Ping-Yuan Lin, Yo-Chia Chen, Han-Hsiang Huang, Wen-Ling Shih

**Affiliations:** 1 Department of Biological Science and Technology, National Pingtung University of Science and Technology, 1, Shuefu Rd., Neipu, Pingtung, 91201 Taiwan; 2 Department of Veterinary Medicine, National Pingtung University of Science and Technology, Pingtung, Taiwan; 3 Department of Veterinary Medicine, National Chiayi University, Chiayi City, Taiwan

**Keywords:** Antibiotic activity, Ribosome-inactivating proteins, MAP30, *Mormodica charantia*

## Abstract

Ribosome-inactivating proteins (RIPs) are a group of enzymes originally isolated from plants that possess the ability to damage ribosomes in an irreversible manner, leading to inhibition of protein synthesis in eukaryotic cells. In this study, we aimed to purify recombinant RIPs, investigate their function in the treatment of bacterial infection, and determine their toxicity in mice. We employed a pMAL protein fusion and purification system using *E. coli* transformed with a plasmid containing MBP-tagged MAP30 cDNA. MBP-tagged MAP30 was purified using a modified novel protocol to effectively produce highly active MAP30 of high purity. In an acute toxicity study in mice, no mortality occurred at doses lower than 1.25 mg/kg. MAP30 at both 0.42 and 0.14 mg/kg induced anti-MAP30 IgG, which reached a maximum titer at week 3. In conclusion, recombinant MAP30 prepared using our purification method possesses bioactivity, and has a synergistic bacteria-killing effect that can significantly reduce the required dosages of chloramphenicol and erythromycin. Therefore, when MAP30 is used in combination with chloramphenicol or erythromycin, it may of benefit in terms of reducing the side effects of the antibiotics, as lower concentrations of antibiotics are required.

## Introduction

Ribosome-inactivating proteins (RIPs) are *N*-glycosidases that were initially isolated from plants. RIPs have potential cytotoxic effects, as they can inhibit protein synthesis by irreversible inactivation of eukaryotic ribosomal RNA (for review see Reyes et al. 2012; Puri et al. 2012). RIPs can be classified into three groups based on their primary structure: type 1 RIPs are single-chain proteins with a molecular weight of about 30 kD; type 2 RIPs are proteins of 60–65 kD that are composed of two chains, an A-chain with rRNA N-glycosidase activity linked to a lectin-like B chain via a disulfide bond; and type 3 RIPs are synthesized as proenzymes and require proteolysis to remove a short internal segment, thus transforming the inactive precursor into an active RIP (Xia et al. 2003; Girbes et al. 2004). In the last 20 years, many researchers have reported that RIPs obtained from different plants exhibit a variety of biological activities, comprising broad spectrum anti-viral (Wang and Tumer 2000), anti-tumor (Sha et al. 2013), and anti-fungal effects through action against fungal ribosomes (Park et al. 2002) and antibacterial (Vivanco et al. 1999), immunosuppressive, embryotoxigenic (Ng et al. 1992), cytotoxic (Battelli 2004) and several enzymatic activities. The most well-known activity of RIPs is inhibition of protein synthesis via *N*-glycosidase action on the eukaryotic 28S rRNA (Peumans et al. 2001). Recently, it has been shown that antimicrobial peptides, such as a cryptic peptide named PDL440-65 (Pizzo et al. 2015), play key roles in the antibacterial activity of RIPs. Additionally, the toxicity of RIPs to animals is highly variable, though a correlation has been established between cytotoxicity and toxicity to animals. Type I and non-toxic type 2 RIPs have a comparable cytotoxicity, which is three orders of magnitude lower than that of toxic type 2 RIPs (Battelli 2004).

*Mormodica charantia* (MC) is a medical plant indigenous to China, and is also widely distributed in Southeast Asia, Africa and certain areas of South America (Fang and Ng 2011). The fruit and seed crude extracts of MC contain large numbers of medicinal components, including cucurbitane-type triterpenoids, linolenic acids, potato- and squash-type protease inhibitors, and type 1 and type 2 RIPs, which have been revealed to exert multiple biological actions (Meng et al. 2012b). MAP30 was first purified from the seeds of MC (Lee-Huang et al. 1990) and has been demonstrated to possess an excellent anti-HIV activity, part of the underlying mechanism of which has been studied. In addition, MAP30 purified from MC has been demonstrated to have a high activity against the herpes simplex virus (HSV) (Bourinbaiar and Lee-Huang 1996), and has been shown to inhibit the proliferation of AIDS-related lymphoma cells infected with herpes simplex virus-8 (HHV-8) by downregulating cellular gene expressions related to uncontrolled proliferation (Sun et al. 2001). It also inhibits the cell proliferation of a panel of tumor cells *in vitro* and *in vivo* (Fang and Ng 2011; Fang et al. 2012a, 2012b). Taken together, previous research has indicated that anti-viral activities are the best-characterized effects of MAP30, especially against HIV.

Although RIPs have been isolated from the seeds of MC, the major ingredient has been identified as α-momorcharin, and the amount of MAP30 is not significant (Meng et al. 2012a). To avoid the inconvenience and impurity experienced when using traditional purification methods, recombinant MAP30 has recently been produced and purified using prokaryotic and eukaryotic systems. The results demonstrated that non-glycosylated MAP30 protein is as active as the glycosylated natural protein, and recombinant MAP30 possesses anti-HIV, anti-HBV, anti-tumor and apoptosis induction activities (Lee-Huang et al. 1995; Fan et al. 2008, 2009). Recent studies have also revealed that MAP30 exerts activities against the herpes simplex virus and pathogenic fungus (Akkouh et al. 2015; Wang et al. 2016), and can induce cell-cycle arrest and apoptosis in human lung carcinoma cells (Fan et al. 2015). However, the effects of recombinant MAP30 in terms of antibacterial activities and possible replacement of antibiotics are unknown, and the toxicity and immunogenicity of MAP30 have also never been studied.

In this study, we developed a new method for purification of insoluble (inclusion body) MAP30 protein, and studied its possible application in the treatment of bacterial infection and its potentiation activity, in addition to evaluating its toxicity in mice.

## Materials and methods

### Plasmid cloning

A pMAL protein fusion and purification system was purchased from NEB (Beverly, MA, USA). Two prokaryotic expression vectors, pMAL-c5X and pMAL-p5X, were utilized to generate plasmids expressing maltose-binding protein (MBP)-fused MAP30 in cytosol and periplasm, respectively. The full-length MAP30 cDNA was isolated from the seeds of *Momordica charantia* using degenerate PCR and confirmed by direct sequencing (kindly provided by Ching-Ming Cheng, Tzu-Chi University, Hualien, Taiwan). The cloned MAP30 genes showed a 99% nucleotide and amino acid identity with the published anti-HIV MAP30 gene (DQ643967). The coding sequence of MAP30 was inserted down-stream from the *malE* gene of *E. coli*. Two plasmids, named c5X-MAP30 and p5X-MAP30, were purified using a maxi plasmid isolation kit (Qiagen, Valencia, CA, USA). Our MAP30 complete sequence was submitted to NCBI (accession number KF745069).

### Modified recombinant protein induction and purification

NEB Express Competent *E. coli* cells (New England Biolabs, Ipswich, MA, USA) were transformed with purified expressing plasmid, and recombinant protein expression was induced by adding IPTG. The transformed bacteria were collected, washed with PBS and lysed with lysis buffer, followed by being subjected to a freeze-thaw cycle four times and ultrasound sonication. The original protocol suggested by the manufacturer used a column buffer (20 mM Tris-HCl, 200 mM NaCl, 1 mM EDTA, 10 mM 2-mercaptoethanol, pH 7.4) for suspension of the cells and washing of the amylose column after the cell lysate was loaded, followed by elution of the bound protein using an elution buffer (column buffer plus 10 mM maltose). The method resulted in a low yield and, therefore, we developed a two-stage purification protocol. In the first stage of our method, cells were lysed in a home-made lysis buffer (including 20 mM Tris-HCl, 200 mM NaCl, 1 mM EDTA, 10 mM 2-mercaptoethanol, pH 7.4, plus 10 μg/ml lysozyme, 1 mM PMSF and 1% Triton X-100). After centrifugation (at 20,000×*g* for 20 min), the soluble fraction was collected by transferring the supernatant into a new tube, and the insoluble fraction (pellet) was solubilized by purification buffer (37 mM NaCl, 2.7 mM KCl, 10 mM Na_2_HPO_4_ and 10 mM NaH_2_PO_4_). The fractions were then loaded into an amylose resin column. After binding, the bound protein was eluted using a modified elution buffer (20 mM Tris-HCl, 200 mM NaCl, 1 mM EDTA, 10 mM 2-mercaptoethanol, pH 7.4 plus 50 mM maltose). In the subsequent further purification process, which aimed to remove the fusion MBP tag, the manufacturer’s method also resulted in a very low protein recovery rate. Therefore, we also developed a new tag removal method, in which the eluted proteins were precipitated by ethanol; the precipitated sample was then incubated with 8 M urea containing 2 mM SDS in PBS for 90 min, and dialyzed against 2 M urea for 30 min at 4 °C. This process effectively separated MBP from MAP30, and MAP30 could then be captured using an anion exchange column and precipitated by ethanol. After centrifugation, MAP30 protein was finally dissolved in GBP buffer (20% glycerol containing 0.5% BSA in PBS) and stored at −20 °C. Samples obtained after each step of the original manufacturer’s protocol and our modified protocol were stored and subjected to SDS-PAGE analysis to compare the yield and recovery rate.

### Assay to examine RIP activity of MAP30

A non-radioactive luciferase assay was utilized to examine the RIP activity of MAP30 (Langer et al. 1996). Briefly, the assay mixture (containing rabbit reticulocyte lysate, reaction buffer supplied by the manufacturer of the lysate, amino acid mixture, RNasin ribonuclease inhibitor, T7 RNA polymerase and T7 control luciferase reporter plasmid) was incubated at 30 °C for 15 min. Then, GBP buffer or various dilutions (100, 10, 1, 0.1 ng/ml) of recombinant MAP30 were added and incubated for an additional 5 min and frozen immediately in liquid nitrogen to stop the translation reaction. After every additional 5 min of incubation, a sample was treated as described above. The luciferase activity was determined by adding luciferase substrate, luciferin (Promega). The value of the control without the addition of MAP30 was used to normalize the other samples, and was set at a 100% translational efficiency. By plotting the luciferase activity versus the MAP30 concentration, the IC_50_ value was calculated using a four-parameter nonlinear regression model.

### Bacterial survival curve and synergism

The effect of MAP30 on the inhibition of bacteria growth was examined using bacterial strains including *Pseudomonas aeruginosa (BCRC10733), Staphylococcus aureus (BCRC 15201), Enterococcus faecalis (BCRC 10789), Salmonella typhimurium (BCRC 12459)* and *Salmonella enteritidis (BCRC 17495).* Based on the ribosome-damaging activity of RIPs, antibiotics acting on 30S or 50S, as well as antibiotics targeting the cell wall, were used in this assay. The minimum inhibitory concentration (MIC) of antibiotics was determined by the serial dilution method, and ranged from 0.512 to 512 μg/ml. 20 μl of bacterial suspension (1.0 × 10^6^ CFU/ml) was added to Brain Heart Infusion (BHI) broth containing antibiotics. After incubation at 37 °C for 24 h, the MIC endpoint was read as the lowest concentration of antibiotics that resulted in no bacterial colony growth in Muller–Hinton agar. All assays were carried out in triplicate. A survival curve for each bacterium was created in order to observe the incubation period necessary for antibiotic antibacterial activity. A synergistic effect between MAP30 and antibiotics was observed following incubation of bacteria with 1/4 or 1/10 of the MIC in the presence of MAP30.

### Measurement of antibiotics inhibition zones

Powders of antibiotics, including ampicillin, tetracycline and kanamycin, were purchased from Sigma (St Louis, MO, USA), and powders of streptomycin, chloramphenicol and erythromycin were obtained from Calbiochem (San Diego, CA, USA). The standard antibiotics concentrations were based on the National Committee on Clinical Laboratory Standards (NCCLS) of the United States. A 6.3-mm paper disk that absorbed 20 μl of antibiotics solution was used for testing. Individual antibiotics concentration for damaging 30S, 50S or cell wall was indicated in Table 1. A single colony of bacteria was selected and inoculated, and grown overnight in Muller–Hinton liquid medium. Inoculates were prepared by diluting the overnight cultures of bacteria suspended in 0.9% NaCl to the level of 0.5 MacFarland standard, and were applied to the plates along with disks containing differing amounts of antibiotics with or without MAP30. We first performed cytotoxicity tests on murine hepatocyte cell line FL83B, and treated the cells with a serial concentration of MAP30 for 48 h. A MTT (3-(4,5-dimethylthiazol-2-yl)-2,5-diphenyltetrazolium bromide) tetrazolium reduction assay was used to calculate the cell viability to obtain the IC_50_ and IC_10_ of MAP30. A synergistic effect between MAP30 and antibiotics was observed following incubation of the bacteria with a serial reduced concentration of antibiotic in the presence of a dose of purified recombinant MAP30 lower than the IC_10_. The criteria for interpretation based upon the diameter of the inhibition zone were according to the NCCLS disk susceptibility test.

**Table 1:** Response patterns of the tested bacteria to antibiotics

Antibiotic (dose)	Ampicillin (10 μg)	Tetracycline (30 μg)	Kanamycin (30 μg)	Streptomycin (10 μg)	Chloramphenicol (30 μg)	Erythromycin (15 μg)
Bacterium						
*S. aureus*	10 (R)	34 (S)	21 (S)	20 (S)	24 (S)	22 (S)
*E. faecalis*	16 (S)	17 (I)	14 (I)	12 (I)	25 (S)	15 (I)
*S. typhimurium*	9 (R)	21(S)	10 (R)	13 (I)	23(S)	10 (R)
*S. enteritidis*	10 (R)	20 (S)	11 (R)	12 (I)	28 (S)	11 (R)
*P. aeruginosa*	8 (R)	12 (R)	10 (R)	11(I)	14 (I)	10 (R)

Standard dosages of the selected antibiotics are shown. The diameter (mm) of the inhibition zone was measured. ‘R’ represents resistance towards antibiotics, ‘I’ represents an intermediate effect, and ‘S’ indicates susceptibility

### Maximum tolerated dosage of MAP30 in mice

The animal test protocol was examined and approved by the IACUC of National Pingtung University of Science and Technology. BALB/c mice of a weight of 25–30 g were randomized into 6 groups, each containing 8 mice, 4 male and 4 female. Purified MAP30 was dissolved in 0.9% NaCl and injected intraperitoneally at doses ranging from 1.25 to 15 mg/kg of body weight. After administration, medical and physical characteristics of the mice, including appearance, mental status, toxic response, and death, were closely observed for 14 days in order to ascertain the maximum tolerated dosage of MAP30.

### Immunogenicity test of MAP30 in mice

The animal test protocol was examined and approved by the IACUC of National Pingtung University of Science and Technology. BALB/c mice of both genders weighing 25–30 g were randomized into 3 groups, 8 mice in each. Based on the previously ascertained maximum tolerated dosage of MAP30, MAP30 at 0.42 and 0.14 mg/kg in 100 μl was injected into the mice in 2 groups, respectively, and 100 μl of normal saline was injected into the mice of the third group as a control. MAP30 was administered via the intraperitoneal route five times every three days. Serum samples were collected before immunization and were taken every 7 days after immunization until 5 blood samples had been obtained. To evaluate the antibody response, purified MAP30 was used to coat 96-well polystyrene plates at 3 μg/well in 50 μl, dried overnight at 37 °C, fixed in 0.2% glutaraldehyde, and then blocked with 3% bovine serum albumin. Serum samples at a series of dilutions were added and incubated at 37 °C for 60 min. HRP-labeled goat anti-mouse IgG and 3′,5,5′-tetramethylbenzidine (TMB) substrate were added to develop the color of the bound substrate. Titers of specific antibodies against MAP30 were measured using an ELISA plate reader.

### Statistical analysis

Data are shown as the mean ± SD. Significance was estimated by the Student *t* test. *P* < 0.05 was considered to indicate statistical significance.

## Results

### Comparison of purification methods between the kit provided and the laboratory-developed modified protocol

**Fig. 1:**
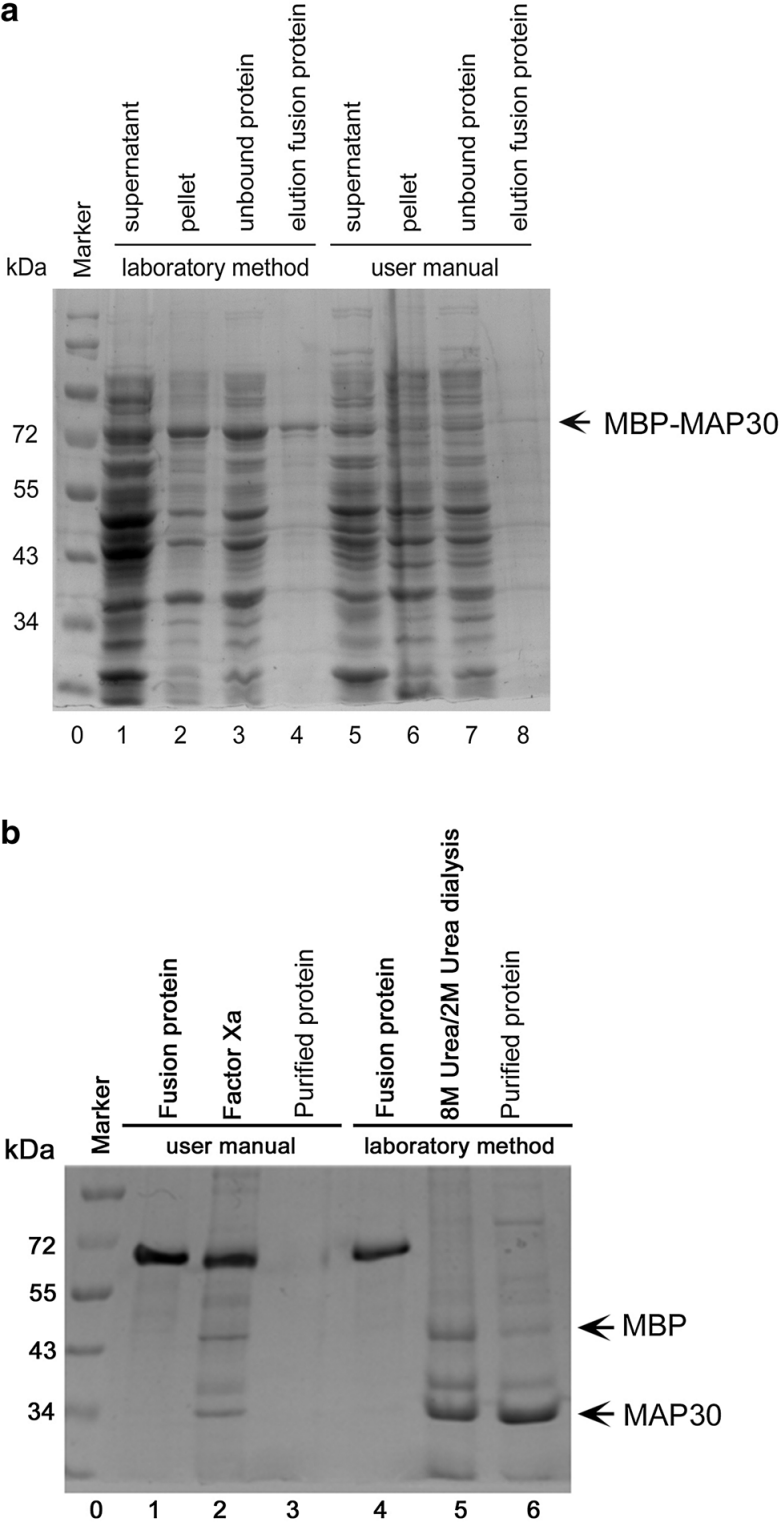
Purification of *E. coli* expressing recombinant MAP30. **a** Phase I purification. *Lanes 1–4* show the results of the new method developed in the laboratory. *Lanes 5–8* show the results of samples processed using the commercial method. Each loading sample was prepared as described in “Materials and methods”. The *arrow* indicates the MBP-MAP30 fusion protein. **b** Phase II purification. Each sample preparation process was performed as described in “Materials and methods”. *Lanes 1–4* show the results obtained following the process described in the commercial user manual. *Lanes 5–8* show the protein purification process followed in our newly developed method. The *two arrows* indicate the MAP tag and MAP30

Two plasmids, c5X-MAP30 and p5X-MAP30, were transformed into NEB Express competent host cells to induce MBP-MAP30 expression in the presence of IPTG. Cultured bacterial samples lysed in two different lysis buffers were compared. Lysis of samples in our home-made lysis buffer (Fig. 1a, lane 1) resulted in more soluble protein being extracted than lysis of samples in the buffer provided by the manufacturer in the kit (Fig. 1a, lane 5). The insoluble pellets were also dissolved in two different purification buffers. Pellets dissolved in our modified buffer had a higher solubilization efficiency than those dissolved in the original buffer provided in the kit (Fig. 1a, lane 2 and lane 6). After amylase resin reaction and elution, our elution buffer resulted in a higher concentration of maltose, indicating a higher fusion protein MBP-MAP30 recovery efficiency (Fig. 1a, lane 4) than that obtained using the original method described by the manufacturer (Fig. 1a, lane 8). To remove the MBP tag, equal amounts of MBP fusion protein obtained previously (Fig. 1a, lane 4) were subjected to further processes to purify the protein (Fig. 1b, lanes 1 and 4). On the other hand, using the method suggested by the manufacturer of NEB, MBP-MAP30 reacted with Factor Xa (Fig. 1b, lane 2), and the MAP tag could not be removed, which resulted in a very low MBP yield (Fig. 1b, lane 3). We inferred that this might be due to a large amount of insoluble protein present in inclusion bodies and, therefore, treated the samples with 8 M urea then dialyzed against 2 M urea, a process that resulted in complete cleavage of MBP (Fig. 1b, lane 5). Samples were then loaded onto amylase resin to remove MBP from MAP30.

### Protein synthesis inhibition activity of purified MAP30 in RIP activity assay

**Fig. 2:**
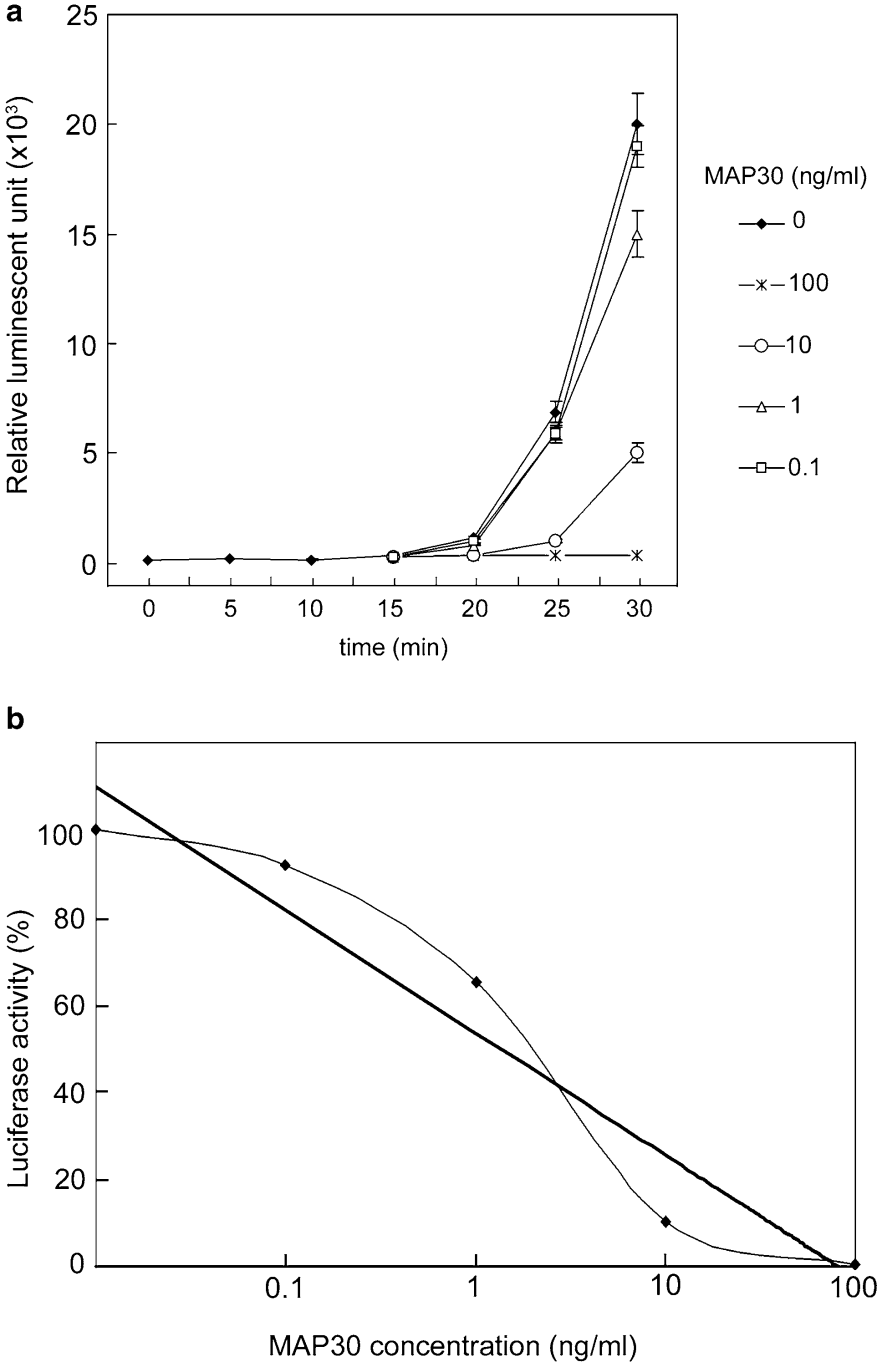
RIP activity assay. **a** Time course of the luciferase activity generated in in vitro translation, measured by luciferin substrate. Various amounts of MAP30 were added as shown. **b** Concentration-dependent reduction of relative luciferase activity by MAP30. The IC_50_ value was obtained from the graph

The time course of luciferase activity generated during in vitro translation was examined to measure the activity of MAP30 at different concentrations. Complete inhibition was observed at 100 ng/ml of MAP30 (Fig. 2a). A plot of the luciferase activity values versus MAP30 concentrations at 30 min revealed that the IC_50_ of MAP30 was 2.29 ng/ml (Fig. 2b). Thus, we established a novel and convenient purification method of MAP30 possessing high RIP activity.

### Synergistic effects on the inhibition of bacterial growth by combinations of MAP30 and 50S-targeting antibiotics

To examine the potential clinical applications of MAP30 in the treatment of infections, five clinically important bacteria strains, including *Staphylococcus aureus*, *Enterococcus faecalis*, *Salmonella typhimurium*, *Salmonella enteritidis*, and *Pseudomonas aeruginosa*, were employed. The MICs of chloramphenicol against each pathogen were determined by the serial dilution method, and were as follows: *S. enteritidis*, 1 µg/ml; *S. aureus*, *E. faecalis* and *S. typhimurium*, all 2 µg/ml; and *P. aeruginosa*, 8 µg/ml.

**Fig. 3:**
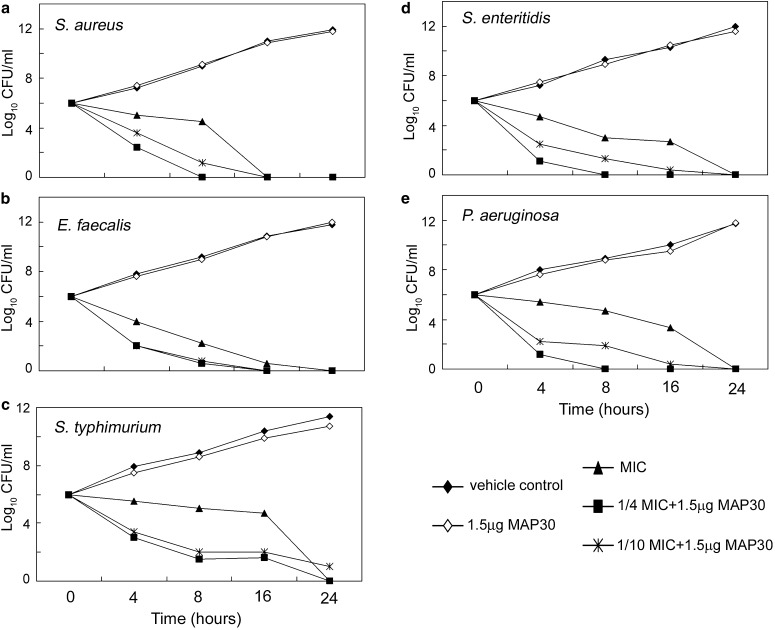
Time course of bacterial growth profile. Chloramphenicol used at 1× the chloramphenicol MIC value, 1/4 of the chloramphenicol MIC value combined with MAP30, 1/10 of the chloramphenicol MIC value combined with MAP30, or MAP30 alone are shown as *different lines*. Colony numbers were counted at different time points during incubation. Examined bacteria isolates are shown. The MIC values of chloramphenicol against each bacterial isolate were as follows: *S. enteritidis*, 1 µg/ml; *S. aureus*, *E. faecalis* and *S. typhimurium*, all 2 µg/ml; and *P. aeruginosa*, 8 µg/ml

We used murine hepatocyte cell line FL83B to perform cytotoxicity testing of MAP30, and ascertained the IC_50_ and IC_10_ of MAP30 to be 11.9 and 1.8 µg/ml, respectively (unpublished data). A dose of 1.5 µg/ml, which is lower than the IC_10_, was chosen for use in this study. In terms of antibiotic activity, MAP30 alone did not exert an antibiotic effect on any of the different bacteria tested (Fig. 3, open diamond). The growth curves of the MICs of chloramphenicol-treated individual bacteria are shown in Fig. 3 (solid triangle). Chloramphenicol at a concentration of 1/4 or 1/10 of the MIC value combined with purified 1.5 μg/ml MAP30 resulted in a synergistic bacteria-killing effect (Fig. 3, solid square and intersection). A similar pattern of change was evident in the case of bacteria treated with erythromycin (data not shown). Thus, MAP30 synergized with two 50S-targeting antibiotics, chloramphenicol and erythromycin, and exhibited a significant bacteria-killing activity. To further assess whether MAP30 has the potential for clinical application, we evaluated the susceptibility of tested bacteria by measurement of inhibition zones. In addition to two 50S-targeting antibiotics, cell-wall inhibitor ampicillin and 30S-targeting antibiotics tetracycline, kanamycin and streptomycin were employed in the assay. First, the individual effect of each antibiotic against each individual bacterium was determined based on the NCCLS criteria [resistant (R), intermediate (I) or susceptible (S)] (Table 1). A reduced antibiotic dosage combined with 1.5 μg recombinant purified MAP30 was also tested. Although the diameter was slightly reduced in the presence of MAP30, the outcome based on the NCCLS criteria did not show an alteration effect in combination with ampicillin, tetracycline, kanamycin or streptomycin (Table 2). Although MAP30 alone did not have an inhibitory effect on bacterial growth, a synergistic effect of MAP30 on the inhibition of bacterial growth was observed when it was used in combination with chloramphenicol or erythromycin (Table 3).

**Table 2:** Response patterns to antibiotics that work against the cell wall and the 30S rRNA subunit are unaltered in the presence of MAP30

Reduced antibiotic + MAP30	Ampicillin (μg)				Tetracycline (μg)				Kanamycin (μg)				Streptomycin (μg)			
	0	1	2	5	0	3	6	15	0	3	6	15	0	1	2	5
	MAP30				MAP30				MAP30				MAP30			
Bacterium																
*S. aureus*	7 (R)	7 (R)	7 (R)	9 (R)	7 (R)	10 (R)	18 (I)	25 (S)	7 (R)	8 (R)	9 (R)	17 (I)	8 (R)	8 (R)	9(R)	17 (I)
*E. faecalis*	6 (R)	8 (R)	10 (R)	12 (I)	8 (R)	10 (R)	16 (I)	16 (I)	7 (R)	7 (R)	8 (R)	13 (I)	6 (R)	9 (R)	9 (R)	14 (R)
*S. typhimurium*	7 (R)	9 (R)	9 (R)	9 (R)	7 (R)	10 (R)	15 (I)	18 (I)	8 (R)	8 (R)	8 (R)	8 (R)	7 (R)	7 (R)	10 (R)	15 (I)
*S. enteritidis*	6 (R)	8 (R)	9 (R)	9 (R)	7 (R)	9 (R)	15 (I)	18 (I)	8 (R)	9 (R)	8 (R)	9 (R)	7 (R)	8 (R)	10 (R)	16 (I)
*P. aeruginosa*	6 (R)	7 (R)	8 (R)	8 (R)	8 (R)	9 (R)	10 (R)	9 (R)	8 (R)	8 (R)	8 (R)	9 (R)	6 (R)	7 (R)	12 (R)	14 (R)

Various reduced antibiotics dosages are shown. Antibiotics were combined with 1.5 μg purified MAP30. The inhibition zone was measured and categorized as ‘R’, ‘I’ or ‘S’

**Table 3:** Synergistic bactericidal effects of antibiotics that work against the 50S rRNA subunit combined with MAP30

Reduced antibiotic + MAP30	Chloramphenicol (μg)				Erythromycin (μg)			
	0	3	6	15	0	3	6	15
	MAP30				MAP30			
Bacterium								
*S. aureus*	7 (R)	24 (S)	26 (S)	32 (S)	7 (R)	22 (I)	26 (S)	30 (S)
*E. faecalis*	7 (R)	21 (S)	25 (S)	26 (S)	7 (R)	16 (I)	23(S)	28 (S)
*S. typhimurium*	7 (R)	24 (S)	26 (S)	28 (S)	8 (R)	19 (I)	23 (S)	26 (S)
*S. enteritidis*	8 (R)	24 (S)	30 (S)	30 (S)	7 (R)	20 (I)	24 (S)	27 (S)
*P. aeruginosa*	8 (R)	16 (I)	19 (S)	22(S)	7 (R)	10 (R)	16 (I)	23 (S)

Various reduced antibiotics dosages are shown. Antibiotics were combined with 1.5 μg purified MAP30. The inhibition zone was measured and categorized as ‘R’, ‘I’ or ‘S’

The results indicated that reduced amounts of 50S-targeting antibiotics combined with MAP30 significantly reduced the inhibition zone diameter and changed the results of antibiotic sensitivity testing based on the NCCLS criteria. With only 3 μg (1/10 of the standard concentration) of chloramphenicol combined with MAP30, the inhibition zone diameter was comparable to that observed with 30 μg chloramphenicol (full standard concentration); 6 μg chloramphenicol combined with MAP30 resulted in a better bacterial inhibition efficiency, and 15 μg chloramphenicol combined with MAP30, half of the standard concentration, revealed a much increased bactericidal activity. In the case of our isolate of *P. aeruginosa*, 30 μg chloramphenicol treatment resulted in an intermediate inhibition level, while a combination of 15 μg chloramphenicol and 1.5 μg MAP30 resulted in a larger inhibition zone diameter, changing the status from intermediate to susceptible (Table 3). Figure 3 also shows that 1/4 or 1/10 of the MIC of chloramphenicol in combination with MAP30 had a remarkable inhibitory effect on all tested bacterial strains. The synergistic effect even reached a level comparable to that of antibiotics alone at the full MICs.

### Maximum tolerated dose of MAP30

It was observed that MAP30 administration resulted in significant acute toxicity. The toxic symptoms included listlessness, lethargy, convulsions and even death. Dead mice were observed in the groups administered doses of 2.5, 5, 10 and 15 mg/kg, while no dead mice were seen in the group administered a 1.25 mg/kg MAP30 injection. The mortality and dead mouse survival duration, as well as the observed symptoms, are shown in Table 4. The LD50 was found to be 3.75 mg/kg by linear regression calculation. The present study showed that the maximum tolerated dose (MTD) of MAP30 was 1.25 mg/kg.

**Table 4:** Mortality induced by intraperitoneal injection of the purified recombinant MAP30

Dose (mg/kg)	Mortality	Survival duration of dead mice (h)	Symptoms
0	0/8 (0%)	Survived	None
1.25	0/8 (0%)	Survived	Salivation, weak, motionless
2.5	2/8 (25%)	16–32	Staggering gait, clonic convulsion
5	6/8 (75%)	8–20	Convulsion, tremor
10	8/8 (100%)	4–12	Severe convulsion, tremor, prone position
15	8/8 (100%)	2–8	Severe convulsion, tremor, prone position

### Immunogenicity of MAP30 in mice

**Fig. 4:**
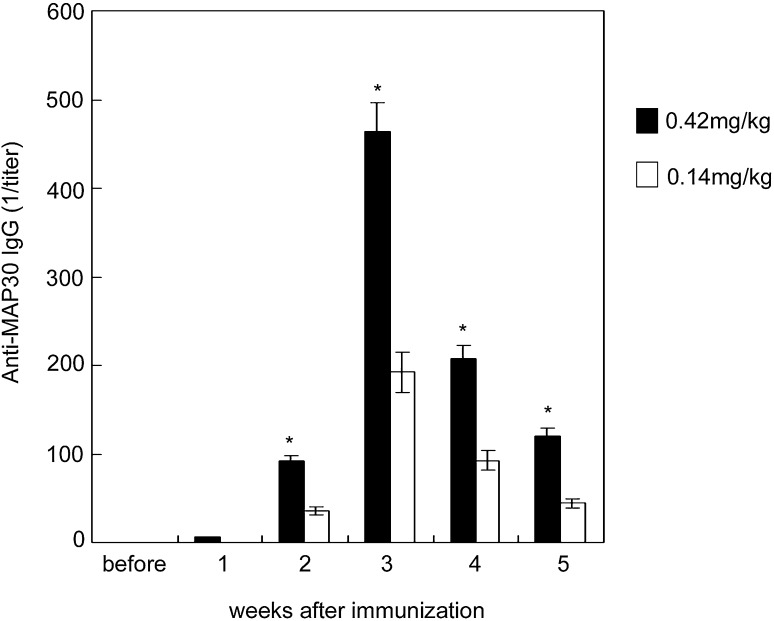
Serum anti-MAP30 titer in mice. Blood was collected via the orbital vein every week after five immunizations. Serum IgG antibody was detected by ELISA and expressed as the mean ± SD. *Asterisks* indicates a significant difference between the immunization and non-immunization groups; *P* < 0.05

Due to the evident toxic symptoms in mice administered MAP30 at a dose higher than 1.25 mg/kg, a reduced amount of MAP30 was used as the immunogen. Figure 4 shows that administration of 0.42 mg/kg (1/3 of the MTD) and 0.14 mg/kg (1/9 of the MTD) through intraperitoneal injection elicited specific antibody production in mice. The antibody titers revealed that the positive response began 2 weeks after immunization, and then reached a peak level at week 3. The titers decreased gradually, but were still obvious at week 5. In the group administered MAP30 at 0.14 mg/kg, the antibody titer showed a weaker positive reaction as compared with the mice administered 0.42 mg/kg.

## Discussion

Because many RIPs have been shown to have interesting pharmacological effects and applications as anti-cancer and anti-viral drugs, RIPs purified from plants have been extensively studied. A key advantage of using recombinant proteins is that large amounts of high-purity proteins can be obtained in a cost-effective way. Recombined MAP30 is expressed in both eukaryotic and prokaryotic cells, and it has been demonstrated that the non-glycosylated form purified from host cells of *E. coli* has a similar activity to that of the glycosylated natural protein (Lee-Huang et al. 1995). MAP30 has been shown to inhibit HIV; it also inhibits the cell proliferation of a panel of tumor cells in vitro and in vivo (Fang and Ng 2011; Fang et al. 2012a, b). Recent studies have also shown that MAP30 can inhibit the herpes simplex virus and pathogenic fungus (Akkouh et al. 2015; Wang et al. 2016), and could induce cell death (Fan et al. 2015). Taken together, the results of previous studies have suggested that the anti-viral activities are the most characterized of MAP30, especially activities against HIV, HBV, and tumor cell proliferation (Lee-Huang et al. 1995; Fan et al. 2008, 2009). In this study, we used recombined MAP30 purified from prokaryotic cells and examined its effects in terms of antibacterial activities when used in combination with different types of antibiotics, and determined its toxicity and immunogenicity.

We found that when the original protocol provided with the commercial pMAL protein purification kit was used, only a very low yield of protein could be recovered from the host bacteria. As overexpressed recombinant proteins are often contained in inclusion bodies and are difficult to isolate by regular protein purification methods (Lilie et al. 1998; De Bernardez Clark 1988), we used a modified protocol, in which detergent and lysozyme were added. Our method resulted in increased extraction and solubility of MAP30 protein from the bacteria. In addition, we also found that the MBP-tag was not able to be removed by Factor Xa treatment provided by the manufacturer of the kit. We, therefore, tested different methods, and found that treatment of the sample with 8 M urea and then dialyzing with 2 M urea effectively separated the MBP-tag from the MAP30 protein.

Several bacterial strains can cause major inflectional diseases. *Enterococcus faecalis* is a Gram-positive, commensal bacterium inhabiting the gastrointestinal tracts of humans and other mammals. *E. faecalis* can cause life-threatening infections in humans, especially in the nosocomial environment, where naturally high levels of resistance to antibiotics are often found, which contribute to its pathogenicity. *Staphylococcus aureus* is Gram-positive, and is frequently found in the respiratory tract and on the skin. The emergence of antibiotic-resistant strains of pathogenic *S. aureus* (e.g., MRSA) is a worldwide problem in clinical medicine. Salmonella consists of a range of very closely related bacteria, many of which cause disease in humans and animals. The three main serovars of *Salmonella enterica* are *S. typhimurium*, *S. enteritidis*, and *S. typhi*. Food poisoning by *Salmonella* species is most commonly due to *S. typhimurium*. *S. enteritidis* has become the single most common serotype that causes food poisoning in the United States (Centers for Disease and Prevention 2013). Epidemic multiple drug-resistant *S. typhimurium* causes invasive disease in sub-Saharan Africa (Kingsley et al. 2009). *Pseudomonas aeruginosa* is a ubiquitous environmental bacterium that is one of the top three causes of opportunistic human infections. Even drug-susceptible strains of *P. aeruginosa* have been shown to have considerable defenses (Livermore 2002; Rex et al. 1995). *P. aeruginosa* carries multiresistance genes, and *P. aeruginosa* has an inducible AmpC β-lactamase that confers resistance to antibiotics (Livermore 2002).

In this pilot study, we examined the possible broad antibacterial function of MAP30, employing representative bacteria including bacteria inhabiting the GI tract, respiratory tract and on the skin, as well as bacteria that cause food poisoning, nosocomial infection and opportunistic human infections. To investigate whether MAP30 has a synergistic effect on antibiotics in clinical application, we first tested its effect on three different types of antibiotic. Of the antibiotics tested, ampicillin belongs to the group of β-lactam antibiotics, which can inhibit cell-wall synthesis; streptomycin, kanamycin and tetracycline bind to the 30S subunit of bacterial rRNA and inhibit protein synthesis (Carter et al. 2000; Brodersen et al. 2000); and chloramphenicol and erythromycin bind to the 50S subunit of rRNA and inhibit protein synthesis via peptidyl transferase inhibition (Levinson 2008). Although our results showed that MAP30 did not alter the activity of antibiotics that either inhibit bacterial cell-wall synthesis or bind to the 30S subunit, we found that with chloramphenicol, MAP30 has a synergistic effect that can significantly reduce the amount of antibiotic required to inhibit bacterial growth. In addition, a lower concentration of erythromycin when combined with MAP30 also resulted in higher bactericidal activity against *E. faecalis* (status changed from intermediate to susceptible), *S. typhimurium* (resistant to susceptible), *S. enteritidis* (resistant to susceptible) and *P. aeruginosa* (resistant to susceptible). Taken together, our results clearly demonstrated that MAP30 changed the response pattern to antibiotics targeting the 50S ribosome, and the purified recombinant MAP30 dramatically reduced the amount of 50S-targeting antibiotics required.

The results of our in vivo study indicated that the LD50 of MAP30 was 3.75 mg/kg in BALB/c mice, and no mortality occurred at a dose of 1.25 mg/ml. As plant proteins, RIPs have strong immunogenicity, as with other native proteins. The immunogenicity of RIPs often becomes a limitation in terms of their use as drugs for therapeutic purposes. In this study, we also investigated the immune response in mice. Our results indicated that continual administration of MAP30 at 0.14 and 0.42 mg/kg induced a peak anti-MAP30 antibody level at 3–4 weeks after the first injection. A high level of anti-MAP30 IgG was detected at a dose 0.42 mg/kg, while when a lower dose of 0.14 mg/kg was used, a high level of anti-MAP30 IgG was only seen during weeks 3 and 4. Chemical modification by dextran or polyethylene glycol (PEG) has been employed to reduce the immunogenicity of RIPs (Zheng et al. 2012; He et al. 1999; Chan et al. 1999). Recently, a study by (Meng et al. 2012b) demonstrated that PEGylated MAP30 has a lower immunogenicity, which may prolong its half-life in vivo. A potential advantage of using recombination proteins is that if a major epitope of an RIP that causes immunogenicity can be identified, it can be easily removed or modified by protein engineering (Chan et al. 2000). A treatment period of 10–14 days, or even shorter, for antibiotic therapy has been recommended (Paul 2006), which suggests that using MAP30 as a synergist in vivo might be feasible only when a short administration period is required in order to exert an optimal antibiotic effect. In addition, if immunogenicity can be reduced, or the half-life of MAP30 can be prolonged, this will improve the potential of application of MAP30 for treatment.

In conclusion, this study developed an effective method by which to prepare recombinant MAP30, and demonstrated that it has a synergistic effect with 50S-targeting antibiotics on the inhibition of bacterial growth. As MAP30 significantly reduced the dosage of chloramphenicol and erythromycin required, a combination of MAP30 with chloramphenicol or erythromycin may be of benefit in terms of reducing the side effects of the antibiotics.
